# Loss of CADM1/TSLC1 Expression Is Associated with Poor Clinical Outcome in Patients with Esophageal Squamous Cell Carcinoma

**DOI:** 10.1155/2016/6947623

**Published:** 2015-12-31

**Authors:** De Zeng, Xiao Wu, Jin Zheng, Yixuan Zhuang, Jiongyu Chen, Chaoquan Hong, Fan Zhang, Mingyao Wu, Danxia Lin

**Affiliations:** ^1^Department of Medical Oncology, Cancer Hospital of Shantou University Medical College, No. 7 Raoping Road, Shantou 515031, China; ^2^Department of Clinical Pathology, Cancer Hospital of Shantou University Medical College, No. 7 Raoping Road, Shantou 515031, China; ^3^Oncology Research Laboratory, Cancer Hospital of Shantou University Medical College, No. 7 Raoping Road, Shantou 515031, China; ^4^Department of Pathology, Shantou University Medical College, No. 22 Xinlin Road, Shantou 515041, China

## Abstract

*Aims*. We sought to determine the relationship between CADM1/TSLC1 expression and clinicopathological characteristics in patients with esophageal squamous cell carcinoma (ESCC) and the correlation with survival.* Materials and Methods*. Two hundred and ninety-three ESCC tissues and paired adjacent normal esophageal tissues were immunohistochemically assessed in this study. The association of CADM1/TSLC1 with clinicopathological parameters, as well as disease-free survival (DFS) and overall survival (OS), was determined based on the Kaplan-Meier method and Cox regression models.* Results*. CADM1/TSLC1 was detected in 236 (80.5%) tumor tissues and 19 (8.0%) paired adjacent normal esophageal tissues. Decreased CADM1/TSLC1 expression was correlated with more advanced histological grade. CADM1/TSLC1 negative tumors were more frequently observed in male cases than in female cases. DFS and OS in the CADM1/TSLC1 negative group were significantly shorter than those in the positive group, particularly in male patients with ESCC.* Conclusion*. Loss or reduction of CADM1/TSLC1 expression is associated with more advanced histological grade and predicts early recurrence and short survival duration. Thus, loss of CADM1/TSLC1 could be a prognostic factor that can be used to assess the risk of recurrence and survival.

## 1. Introduction

The mortality and morbidity associated with esophageal cancer (EC) remain high throughout the world. Chaoshan region of southern China has a notable high burden, with an age-standardized incidence of approximately 100/100,000 people in Nanao Island [1], a rate that is 5 times higher than that of surrounding areas and 8 times higher than that for the rest of China [2]. Despite advances in surgical techniques and multimodality therapies, the 5-year survival rate remains dismal (14%); additionally, the majority of patients present at an advanced stage, resulting in poor treatment response and prognosis [3].

Esophageal squamous cell carcinoma (ESCC) is the principal histological subtype in China and accounts for more than 90% of all EC [4]. Accumulating studies have focused on identifying biomarkers for early detection and prognostic prediction of ESCC, as well as finding promising molecular therapeutic targets for treatment. Several genetic alterations associated with development and progression of ESCC have been described, including TP53, cyclin D1, E-cadherin, Bcl-2, TNF-alpha, NF-kappaB, TGF-beta, MMP-7, COX-2, EGFR, HER2/neu, and HIF-1 alpha [5]. Nonetheless, only a few of these genes have been demonstrated to be associated with biological or pathological features of ESCC. Therefore, identification of novel genes associated with the development and progression of ESCCs is urgently needed.

Cell adhesion molecule 1 (CADM1/TSLC1), belonging to the immunoglobulin superfamily, is involved in normal cell adhesion, proliferation, and differentiation and plays an important role in the suppression of malignant tumor cell invasion and metastasis [6–8]. It has been reported that the deactivation of the CADM1/TSLC1 gene, partly through promoter hypermethylation, is associated with the occurrence and development of a wide variety of tumors, including non-small cell lung cancer, ovarian cancer, breast cancer, colon cancer, and laryngeal squamous cell carcinoma [9–13].

Ito and colleagues reported that loss of CADM1/TSLC1 expression plays an important role in tumor growth, cell motility, and invasion and is associated with aggressive tumor behavior in ESCC [14]. However, data on the role of CADM1/TSLC1 in ESCC progression is limited. In particular, the clinical significance and function mediated by TSLC1 in ESCC are underinvestigated. In this study, we analyzed the expression of CADM1/TSLC1 using immunohistochemical methodology in ESCC and paired adjacent normal esophageal tissue from patients with EC in Chaoshan region and examined its relationship to clinical and pathological parameters.

## 2. Materials and Methods

### 2.1. Ethical Statement

This study was approved by the Ethics Committee of Cancer Hospital of Shantou University Medical College and was conducted according to the principles expressed in the Declaration of Helsinki [15]. Written informed consent was obtained from all surgical patients to use resected samples for research.

### 2.2. Patient Information and Reagents

ESCC tissue specimens and paired adjacent normal esophageal tissues were obtained from 293 patients (median age, 59.7 years, and range 38–80 years) who underwent surgery in the Cancer Hospital of Shantou University Medical College from November 2001 to June 2004. All the patients had received surgery without previous radiotherapy or chemotherapy and had no evidence of metastasis at the first visit. All the tumors were confirmed as ESCCs by pathologists in Clinical Pathology Department of Shantou University Medical College and were classified according to the 7th edition of the tumor-node-metastasis (TNM) classification of the International Union against Cancer (UICC). Patients were included in this study only if a follow-up was obtained. Patients' data are summarized in Table 1.

Rabbit polyclonal antibody (ab3910) to CADM1/TSLC1, general EnVision kit, and DAB chromogenic reagent kits were used in the study, all purchased from Abcam company (Abcam, MA, USA).

### 2.3. Immunohistochemistry

Formalin-fixed and paraffin-embedded tissues were cut into 4-micron-thick sections, stained with haematoxylin and eosin (H&E). Histological classification was determined by two pathologists according to the 2003 World Health Organization Criteria. In the negative controls, primary antibodies were omitted and replaced by PBS. And 3% hydrogen peroxide was used for 30 minutes to inactivate endogenous peroxidase activity. Thereafter, sections were treated with peroxidase-conjugated goat anti-rabbit or anti-mouse antibodies. Counterstaining was carried out using haematoxylin. Results were independently evaluated by two investigators without prior knowledge of patient information. Sections were visualized under a bright-field microscope (Olympus, Japan), and staining intensity and subcellular localization were evaluated twice in a blinded manner based on a preagreed staining scoring standard from specialized pathologists.

For cytoplasmic CADM1/TSLC1 expression, no expression, weak expression, moderate expression, or strong expression was recorded as 0, 1, 2, and 3 for staining intensity, and the percentage of positive cells was also scored as 5 categories, 0 for <10%, 1 for 1–25%, 2 for 26–50%, 3 for 51–75%, and 4 for >75%. In the cases with a discrepancy between duplicated cores, the average score from the two tissue cores was taken as the final score. Agreement between the two evaluators was 95%, and all scoring discrepancies were resolved through discussion between the two evaluators. The levels of CADM1/TSLC1 staining were calculated by multiplying the scores of staining intensity and the percentage of positive cells to define negative expression (0 score) and positive expression (≥1 score).

### 2.4. Follow-Up and Statistical Analysis

The overall survival (OS) time was defined as the time from the date of diagnosis to the death of any cause of the patient or the last follow-up visit. The disease-free survival (DFS) was from the date of diagnosis to the date of relapse. In this cohort, overall survival (OS) ranged between 1 month and 129 months, and the median OS was 48.2 months. At the end of the study, 220 esophageal cancer-specific deaths and 219 esophageal cancer relapses were observed. Disease-free survival (DFS) ranged from 1 month to 129 months, with a median DFS of 36.8 months.

Levels of statistical significance were evaluated with data by using the chi-square test. The Spearman rank correlation coefficient was used for ordinal variables. Logistic regression was used to examine the association between CADM1/TSLC1 expression and the various clinicopathological parameters. Disease-free survival (DFS) and overall survival (OS) were calculated with the Kaplan-Meier method and the differences in survival were determined by log-rank test. All statistical differences were considered significant at the level of *p* < 0.05. All data were analyzed with SPSS 19.0 for Windows.

## 3. Results

### 3.1. Expression of CADM1/TSLC1 in ESCC and Pared Adjacent Normal Esophageal Tissues

**Figure 1:**
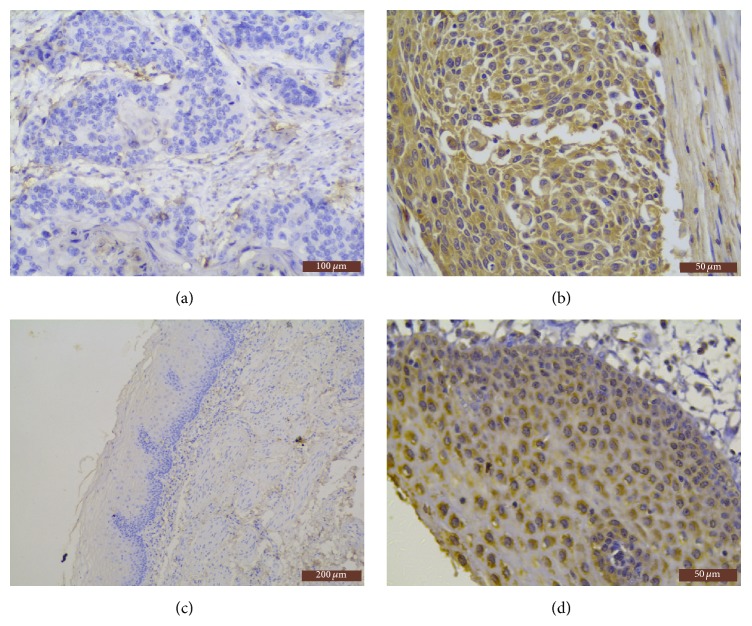
Representative immunohistochemical staining results for CADM1/TSLC1 in tissues of ESCC and normal esophageal tissues. (a) Negative expression of CADM1/TSLC1 in ESCC tissues (SP ×200 magnification); (b) positive expression of CADM1/TSLC1 in ESCC tissues (SP ×400 magnification); (c) negative expression of CADM1/TSLC1 in normal esophageal tissues (SP ×100 magnification); (d) positive expression of CADM1/TSLC1 in normal esophageal tissues (SP ×400 magnification).

CADM1/TSLC1 mainly was expressed in the cytoplasm of esophageal squamous cancer cells. The cytoplasmic positive cells were showing IHC staining as tan granular or patchy coloring (Figure 1(b)). There were 236 cases (80.5%) with CADM1/TSLC1 positive expression in 293 cases of esophageal squamous cell carcinoma (ESCC), with 1 score in 20 cases, 2 scores in 41 cases, 3 scores in 41 cases, 4 scores in 93 cases, and 6 scores in 41 cases, respectively, and 57 cases were CADM1/TSLC1 negative expression (Figure 1(a)).

Most of CADM1/TSLC1 positive cells were showing IHC staining as tiny particles diffusing in the cytoplasm in normal esophageal tissues (Figure 1(d)). In 293 cases with nonneoplastic esophageal tissues, 19 cases were CADM1/TSLC1 positive, and the positive rate was 8.0%, with 1 score in 17 cases and 2 scores in 2 cases. Positive expression of CADM1/TSLC1 in ESCC was significantly higher than that of nonneoplastic esophageal tissues (*p* < 0.01). And there was no positive expression in blank control group (Figure 1(c)).

### 3.2. Clinicopathological Analysis of CADM1/TSLC1 Expression in ESCC

CADM1/TSLC1 positive tumors were more frequently observed in male cases (171/204 cases, 83.8%) than in female cases (65/89 cases, 73.0%) (*p* < 0.05). And the positive rate of CADM1/TSLC1 was associated with the tumor pathological grade (*p* < 0.01), with grade I, grade II, and grade III being 86.5% (77/89), 89.0% (145/163), and 34.1% (14/41), respectively, and the difference was statistically significant (*p* < 0.01). CADM1/TSLC1 expression was not associated with age, location of tumor, depth of infiltration, tumor size, lymph node metastasis, or clinical stage (*p* > 0.05), as shown in Table 1.

### 3.3. Loss of CADM1/TSLC1 Expression in ESCC Is Associated with Poor DFS and OS of Patients with ESCC

There were 293 cases of postoperative patients included for follow-up analysis with the last follow-up visit in July 2014. There were 38 patients still alive and 36 patients lost to follow-up. In the 219 patients with ESCC who died during the follow-up period, the shortest survival time was 1 month, and the longest survival time was 129 months. Among the 219 deaths included in the survival analysis, 199 cases were caused by ESCC recurrence or distant metastasis, and 20 cases were not cancer-related. In 142 patients with ESCC distant metastasis, 80 cases underwent palliative chemotherapy and 14 cases did not receive any treatment. There were 48 cases lacking sufficient information of subsequent treatment after distant metastasis. Fifty-seven cases with local recurrence or regional lymph node involvement received concurrent palliative radiotherapy and chemotherapy.

**Figure 2:**
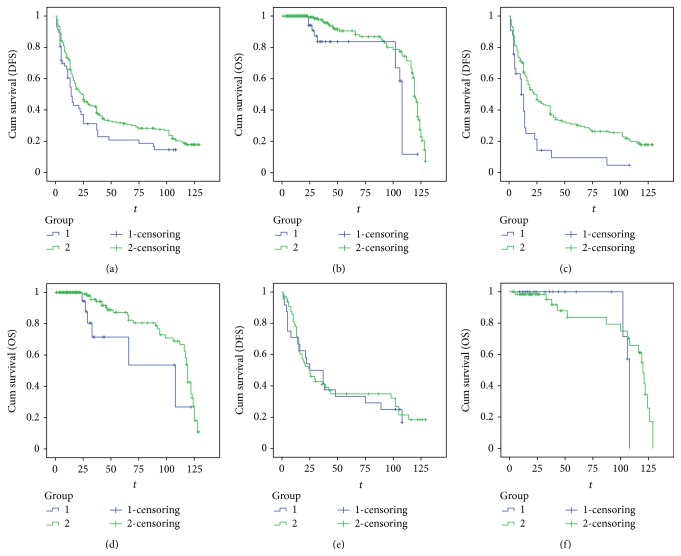
Relationship between DFS or OS and CADM1/TSLC1 expression. (a) Relationship between DFS and CADM1/TSCL1 expression in all patients with ESCC; (b) Kaplan-Meier curves for the correlation between OS and CADM1/TSCL1 expression in all patients with ESCC; (c) Kaplan-Meier curves for the correlation between DFS and CADM1/TSCL1 expression in male patients with ESCC; (d) Kaplan-Meier curves for the correlation between OS and CADM1/TSCL1 expression in male patients with ESCC; (e) Kaplan-Meier curves for the correlation between DFS and CADM1/TSCL1 expression in female patients with ESCC; (f) Kaplan-Meier curves for the correlation between OS and CADM1/TSCL1 expression in female patients with ESCC.

The median DFS of CADM1/TSLC1 positive expression group and negative expression was 24.0 (95% CI: 18.1 to 29.9) months and 14.0 (95% CI: 10.6 to 17.4) months, respectively. And there was statistical difference between these two groups (*p* = 0.047) (Figure 2(a)). The median overall survival of CADM1/TSLC1 positive and negative expression group was 119.0 (95% CI: 117.1 to 120.9) months and 108.0 (95% CI: 107.1 to 108.9) months, respectively. The comparison between CADM1/TSLC1 positive and negative groups was statistically different (*p* < 0.01) (Figure 2(b)).

### 3.4. The Correlation between CADM1/TSLC1 Expression and Patient Survival Is Affected by Sex

Subgroup analysis was carried out to investigate the effect of sex on the survival of ESCC patients with positive or negative CADM1/TSLC1 expression. The Kaplan-Meier curve and log-rank test were applied to each sex group. We found that negative expression of CADM1/TSLC1 in male patients was associated with poor DFS (log-rank = 12.609, *p* = 0.000) (Figure 2(c)) and OS (log-rank = 3.965, *p* = 0.046) (Figure 2(d)). However, in female patients, CADM1/TSLC1 expression did not affect either DFS (log-rank = 0.056, *p* = 0.813) (Figure 2(e)) or OS (log-rank = 1.792, *p* = 0.181) (Figure 2(f)). In the CADM1/TSLC1 positive group, there was no significant sexual difference either in DFS or in OS.

## 4. Discussion

CADM1/TSLC1, also called IGSF4-3, was first identified by Murakami in the study of lung adenocarcinoma A549 cell lines through functional complementary methods for determining new tumor-suppressor gene [16, 17]. Previous studies have found that CADM1/TSLC1 expression was absent or reduced in a variety of malignant tumors and was associated with growth and invasion in malignancy [18]. Liang and colleagues found that CADM1/TSLC1 gene could inhibit the proliferation of esophageal cancer cell line Eca109 and block the transition from G1 phase to S phase in cell cycle, thus inducing apoptosis [8]. Furthermore, Lee and colleagues reported that cohypermethylation of CADM1 in combination with p14 may be an independent prognostic factor for recurrence in patients with stage I ESCC [19].

Our study demonstrated that the majority of esophageal squamous carcinoma (ESCC) expressed CADM1/TSLC1 and the positive rate were obviously higher than those in adjacent normal esophageal tissues, as well as higher than those in other studies of CADM1/TSLC1 in ESCC. For our consideration, the discrepancy may reflect the distinct study population with different genetic background and environmental exposure in Chaoshan region or alternatively may also arise from several sources, for example, tumor grade, antibody efficiency, scoring method, and other methodological aspects related to the IHC.

Our study also showed that CADM1/TSLC1 expression was significantly associated with sex and tumor pathological grade, in which CADM1/TSLC1 was more frequently expressed in male patients than in female patients with ESCC. And positive expression of CADM1/TSLC1 was evidently higher in well-differentiated ESCC than that in the poor-differentiated ESCC, and the results were consistent with previous reports by other investigators [16]. Presumably, CADM1/TSLC1 might act as a pivotal suppressing force in the malignant transformation of ESCC. However, there was no significant correlation observed between expression of CADM1/TSLC1 and age, tumor location, depth of tumor invasion, tumor size, lymph node metastasis, or clinical stage.

In Kaplan-Meier survival analysis, we found that the overall survival (OS) of CADM1/TSLC1 negative group was significantly lower than that in CADM1/TSLC1 positive group, which was consistent with the results in other studies of esophageal squamous cell carcinoma (ESCC) [14], as well as skin squamous cell carcinoma [20], laryngeal carcinoma [13], meningioma [21], breast cancer [22], nasopharyngeal carcinoma (NPC) [23], lung adenocarcinoma [9], and colon cancer [12]. Furthermore, our results suggested that CADM1/TSLC1 negative expression was associated with shorter disease-free survival (DFS) and accordingly might predict early relapse of patients with ESCC after surgery.

Interestingly, in the subgroup analysis, it is noteworthy that male patients with positive expression of CADM1/TSLC1 had a longer DFS and higher survival probability than those with negative expression of CADM1/TSLC1. In contrast, the expression of CADM1/TSLC1 did not influence DFS or OS in female patients. Our findings support the viewpoint of the study on lung adenocarcinoma that the prognostic impact of loss of CADM1/TSLC1 was significant for male patients but not for female patients [4], since it has been widely recognized that the prognosis of men and women with ESCC is distinct and TSLC1 might be one of the genes that play a different role in men and women with ESCC.

The drawback of the study is that the expression of CADM1/TSLC1 in recurrence tissues was not provided, primarily due to low biopsy rate in patients with recurrent esophageal carcinoma. And all the cases were collected from a single institution, which might compromise the representativeness of the population in this region. Further study is warranted and might focus on evaluating the potential role and precise mechanism of CADM1/TSLC1 in the signaling pathway of pathogenesis, invasion, and metastasis of esophageal squamous carcinoma (ESCC).

## 5. Conclusion and Future Perspective

In summary, CADM1/TSLC1 was detected in the majority of ESCC tissues and the loss or reduction of TSLC1 expression was associated with gender and tumor pathological grade. CADM1/TSLC1 negative expression could predict early recurrence and short survival duration, particularly in men with ESCC. It is promising to become an important diagnostic and prognostic biomarker, as well as a potential target for gene therapy of esophageal cancer.

## Figures and Tables

**Table 1:** Relationship between CADM1/TSLC1 expression and clinicopathological variables of ESCC.

Variable	CADM1/TSLC1 expression		*p* value
	Positive (*n* = 236)	Negative (*n* = 57)	
Age (years)			0.145
<60	128 (54.2%)	37 (64.9%)	
≥60	108 (45.8%)	20 (35.1%)	
Sex			0.032
Male	171 (72.5%)	33 (57.9%)	
Female	65 (27.5%)	24 (42.1%)	
Location			0.674
Upper thoracic	25 (10.6%)	4 (7.0%)	
Middle thoracic	179 (75.8%)	46 (80.7%)	
Lower thoracic	32 (13.6%)	7 (12.3%)	
Histological grade			0.000
I	77 (32.6%)	12 (21.1%)	
II	145 (61.4%)	18 (31.6%)	
III	14 (6.0%)	27 (47.3%)	
Depth of invasion			0.791
T1-2	46 (19.5%)	12 (21.1%)	
T3-4	190 (80.5%)	45 (78.9%)	
Tumor size (cm)			0.410
≤5	165 (70.0%)	43 (75.4%)	
>5	71 (30.0%)	14 (24.6%)	
Lymph node metastasis			0.997
Positive	116 (49.2%)	28 (49.1%)	
Negative	120 (50.8%)	29 (50.9%)	
TNM stage			0.905
I-II	118 (50.0%)	28 (49.1%)	
III	118 (50.0%)	29 (50.9%)	
